# Supplementary material for: Content-rich biological network constructed by mining PubMed abstracts
Source: BMC Bioinformatics. 2004 Oct 8;5:147. doi: 10.1186/1471-2105-5-147 (PMC528731; doi:10.1186/1471-2105-5-147)
Supplement: Additional File 2 — The original results of the above study (non-essential files are deleted to keep the file size under the limit set by BMC bioinformatics). [file 1471-2105-5-147-S2.bz2 › chilibotAdditionalFile2/dip20/568ID8386828E10932/html/FGF5_FGFR1.html]

 


 **FGF5** and **FGFR1** 
  
Found 20 abstracts in PubMed, retrieved 20.  
 

 What does Google say? 
 PDF only 
| .edu only 

---

**Interactive relationship** (e.g. stimulation, inhibition, etc)

**Neutral relationship**- Competition binding studies show that the KD for FGF 5  [ **FGF5** ]  FGFR 1  [ **FGFR1** ]  and FGF 5  [ **FGF5** ]  FGFR  [ **FGFR1** ]  2 interactions are both between 0.5 and 1.5 x 10 9 M.  Ref: 8386828 Oncogene, 1993
- The temporal sequence of FGF 5  [ **FGF5** ]  mRNA expression was similar to that of FGFR  [ **FGFR1** ]  4 mRNA expression.  Ref: 9221924 Brain Res Mol Brain Res, 1997
- FGF 5  [ **FGF5** ]  can bind and induce autophosphorylation of human FGF receptors FGFR 1  [ **FGFR1** ]  and 2.  Ref: 8386828 Oncogene, 1993
- The distinctive pattern of the FGFR 1  [ **FGFR1** ]  mRNA response appears to indicate that FGFR 1  [ **FGFR1** ]  is a factor in the modulation of the cellular response for FGF 5  [ **FGF5** ]  and FGF 2.  Ref: 7649223 Exp Neurol, 1995
- FGF 5  [ **FGF5** ]  is a secreted FGF that is believed to preferentially act via the IIIc splice variant of FGFR 1  [ **FGFR1** ] .  Ref: 11876253 Int J Pancreatol, 2001
- In contrast, TAKA 1 clones expressing FGFR 1  [ **FGFR1** ]  IIIc were growth stimulated in the presence of FGF 5  [ **FGF5** ]  and displayed enhanced mitogen activated protein kinase MAPK activity in the presence of FGF 5  [ **FGF5** ] .  Ref: 11876253 Int J Pancreatol, 2001

**Non-interactive relationship** (e.g. studied together, co-existance, homology, etc.)

- AIM In this study we investigated the importance of FGFR 1  [ **FGFR1** ]  IIIc expression for FGF 5  [ **FGF5** ]  mitogenic signaling in a pancreatic ductal cell line.  Ref: 11876253 Int J Pancreatol, 2001
- CONCLUSION Our data demonstrate that FGFR 1  [ **FGFR1** ]  IIIc can mediate FGF 5  [ **FGF5** ]  induced mitogenesis via the MAPK pathway in pancreatic ductal cells, and suggest that expression of FGFR 1  [ **FGFR1** ]  IIIc in conjunction with FGF 5  [ **FGF5** ]  may contribute to the pathobiology of human pancreatic cancer.  Ref: 11876253 Int J Pancreatol, 2001
- A cDNA encoding FGFR 1  [ **FGFR1** ]  IIIc was stably transfected into the well differentiated TAKA 1 pancreatic ductal cell line that is not responsive to  **FGF5**  and does not express FGFR 1  [ **FGFR1** ] .  Ref: 12105858 Gastroenterology, 2002
- As the proliferation of granule cells in the external granule layer and their migration through the molecular layer into the internal granule layer actively occur during these periods, the present findings suggest that FGF 5  [ **FGF5** ]  as well as FGFR  [ **FGFR1** ]  4 might play important roles in the proliferation and or migration of granule cells during the post natal development of the cerebellar cortex.  Ref: 9221924 Brain Res Mol Brain Res, 1997
- RESULTS We detected the expression of mRNAs for FGF1 through  **FGF5** , FGF7 through FGF10, and  **FGFR1**  through FGFR4 IIIb and IIIc splice variants in rat metanephroi from E14 to E21.  Ref: 10594778 Kidney Int, 1999
- Given that FGF 5  [ **FGF5** ]  receptor  **FGFR1**  is expressed in dermal papilla cells DPCs, which are known to stimulate outer root sheath cell ORSC proliferation, we hypothesized that FGF 5  [ **FGF5** ]  attenuates DPC mediated ORSC proliferation.  Ref: 11779149 Biochem Biophys Res Commun, 2002
- RESULTS TAKA 1 cells secrete FGF 5  [ **FGF5** ] , but were found not to express FGFR 1  [ **FGFR1** ]  and to be unresponsive to exogenous FGF 5  [ **FGF5** ] .  Ref: 11876253 Int J Pancreatol, 2001
- These results demonstrate that brain activity exerts influences at the gene expression levels of FGFR 1  [ **FGFR1** ]  and its ligands FGF 5  [ **FGF5** ]  and FGF 2.  Ref: 7649223 Exp Neurol, 1995
- Epileptiform activity was induced by kainic acid injection, and in situ hybridization was used to assess the progress of changes in distribution and intensities of FGF receptor 1 FGFR 1  [ **FGFR1** ] , FGF 5  [ **FGF5** ] , and FGF 2 mRNAs.  Ref: 7649223 Exp Neurol, 1995
- Human pancreatic ductal carcinoma cells express FGF 5  [ **FGF5** ]  and FGFR  [ **FGFR1** ]  1IIIc, implying a potential for autocrine growth modulation.  Ref: 11876253 Int J Pancreatol, 2001
